# Modified-ramped position: a new position for intubation of obese females: a randomized controlled pilot study

**DOI:** 10.1186/s12871-020-01070-2

**Published:** 2020-06-17

**Authors:** Ahmed Hasanin, Hager Tarek, Maha M. A. Mostafa, Amany Arafa, Ahmed G. Safina, Mona H. Elsherbiny, Osama Hosny, Ahmed A. Gado, Tarek Almenesey, Ghada Adel Hamden, Mohamed Mahmoud, Sarah Amin

**Affiliations:** 1 Department of anesthesia and critical care medicine, Cairo university, Giza, Egypt; 2 Department of surgery, Cairo university, Giza, Egypt; 3 Department of anesthesia and critical care medicine, Beni suef university, Beni suef, Egypt

**Keywords:** Obese, Female, Laryngoscopy, Ramped position, Modified-ramped position

## Abstract

**Background:**

Endotracheal intubation requires optimum position of the head and neck. In obese females, the usual ramped position might not provide adequate intubating conditions. We hypothesized that a new position, termed modified-ramped position, during induction of anesthesia would facilitate endotracheal intubation through bringing the breasts away from the laryngoscope and would also improve the laryngeal visualization.

**Methods:**

Sixty obese female patients scheduled for general anesthesia were randomly assigned into either ramped or modified-ramped position during induction of anesthesia. In the ramped position (*n =* 30), the patient head and shoulders were elevated to achieve alignment of the sternal notch and the external auditory meatus; while in the modified-ramped position (*n =* 30), the patient shoulders were elevated using a special pillow, and the head was extended to the most possible range. Our primary outcome was the incidence of failed laryngoscopic insertion in the oral cavity (the need for patient repositioning). Other outcomes included time till vocal cord visualization, time till successful endotracheal intubation, difficulty of the mask ventilation, and Cormack-Lehane grade for laryngeal view.

**Results:**

Fourteen patients (47%) in ramped group required repositioning to facilitate introduction of the laryngoscope in the oral cavity in comparison to one patient (3%) in the modified-ramped position (*p <* 0.001). Modified-ramped position showed lower incidence of difficult mask ventilation, shorter time for glottic visualization, and shorter time for endotracheal tube insertion compared to the ramped position. The Cormack-Lehane grade was better in the modified-ramped position.

**Conclusion:**

Modified-ramped position provided better intubating conditions, improved the laryngeal view, and eliminated the need for repositioning of obese female patients during insertion of the laryngoscope compared to ramped position.

**Clinical trial registration:**

Identifier: NCT03640442. Date: August 2018.

## Background

Adequate conditions for endotracheal intubation require appropriate positioning of head and neck. The most appropriate position for laryngeal visualization, termed “sniffing position” [1], requires flexion of the neck by 35° (achieved by head elevation), and extension of the head by 15° [2] to have the sternum at the same level of the external auditory meatus [3, 4]. Sniffing position maintains the alignment of the three axes, namely the oral, the pharyngeal, and the laryngeal axes, to reach the optimal laryngeal visualization [1]. In patients with obesity, the ramped position was suggested to achieve better intubating conditions [3, 5]. However, the data for the optimum position for intubating patients with obesity are conflicting [3, 5, 6]. Semler et al. pointed out that putting patients in the ramped position increased the numbers of intubation trials through wide-ranging of body mass indices [6]. Thus, it had been suggested that more research and modifications are warranted to reach the proper intubating position [7, 8].

**Fig. 1:**
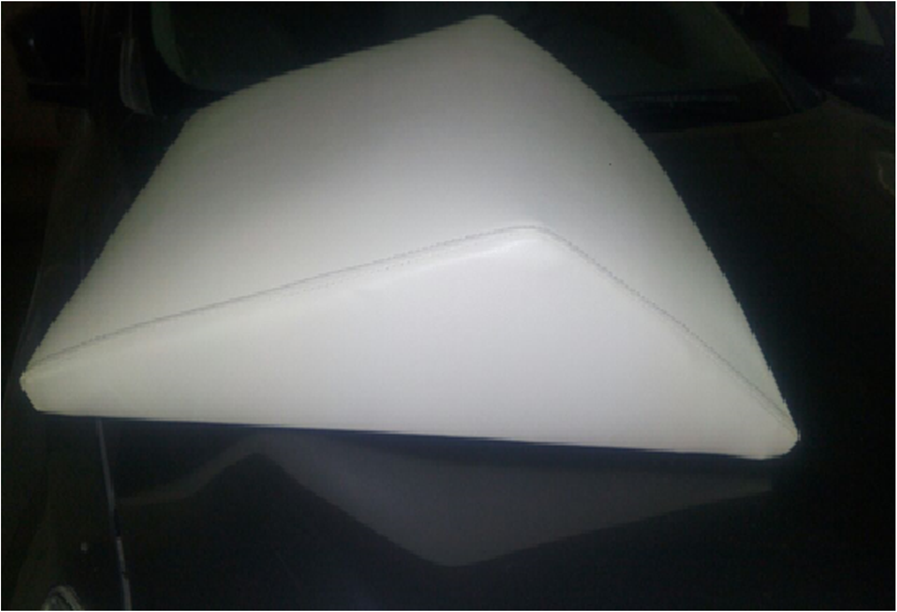
The special pillow designed for achieving modified ramped position (Hasanin Pillow)

In obese females, laryngoscopy is usually impeded by patients’ breasts; therefore, the intubation process could be prolonged leading to serious hypoxia [9]. We hypothesized that using a special pillow (Fig. 1) to achieve a modified-ramped position, through slight neck extension than that offered in the ramped position, and more head extension, would improve the intubating conditions in obese females. We hypothesized that this slight head, and neck extension at the beginning of the laryngoscopy would bring the breasts away from the laryngoscope and would also improve the laryngeal visualization. The aim of this pilot study was to investigate the feasibility of using the modified-ramped position for laryngoscopy in obese females compared to the traditional ramped position.

## Methods

This randomized controlled study was conducted in Cairo University Hospital after institutional board review approval (N-107-2018) from September 2018 till February 2019. The study was registered before recruitment of the first participant at clinicaltrials.gov registry system on 21 August 2018 (NCT03640442). Written informed consents were obtained from all participants before enrollment. Randomization was achieved using computer-generated sequence. Concealment was achieved using opaque, closed envelopes by a research assistant who had no further involvement in the study.

The study included: obese female patients (body mass index above 35 kg/m^2^), aged above 18 years, with American Society of Anesthesiologists class II or III, scheduled for any operation under general anesthesia with endotracheal intubation. Patients with facial or neck scars, edentulous patients, patients with unstable cervical spine, patients with limited neck extension and patients with airway masses were excluded. Five patients were excluded for the following reasons: edentulous (1 patient), limited neck extension (2 patients), refusal to participate (2 patients).

On arrival to the operating room, airway assessment for the patients was performed (Mallampati score, thyromental distance, mouth opening, and neck extension). Patients received the routine preoperative medications (metoclopramide 10 mg intravenous and ranitidine 50 mg intravenous). Routine monitors, including electrocardiogram, non-invasive blood pressure monitor, and pulse oximetry were applied before induction of anesthesia. End-tidal capnography was applied after endotracheal intubation. Before induction of anesthesia, patients were randomized to be initially settled into either ramped group (*n =* 30) or modified ramped group (*n =* 30).

### Details of each position

**Fig. 2:**
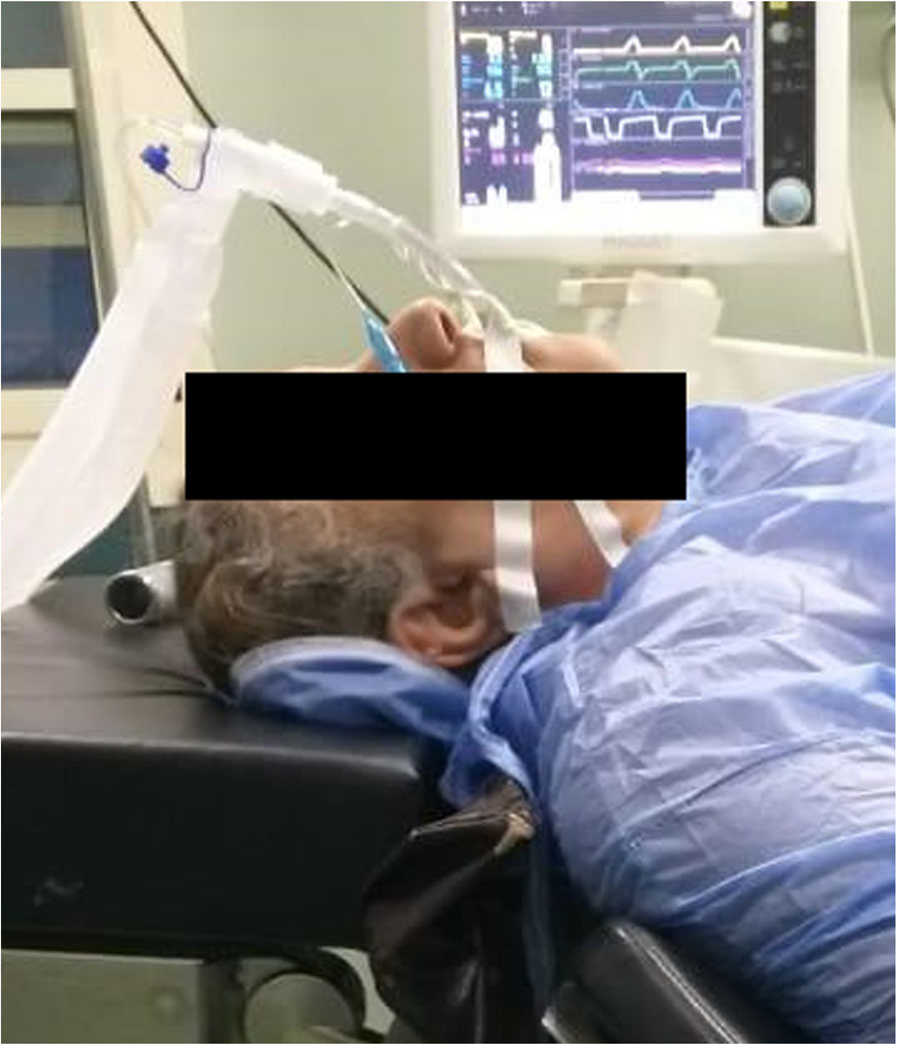
The ramped position

Ramped position: This position was achieved by elevation of the shoulders and the head till achieving alignment of the sternal notch and the external auditory meatus (as shown in Fig. 2).

**Fig. 3:**
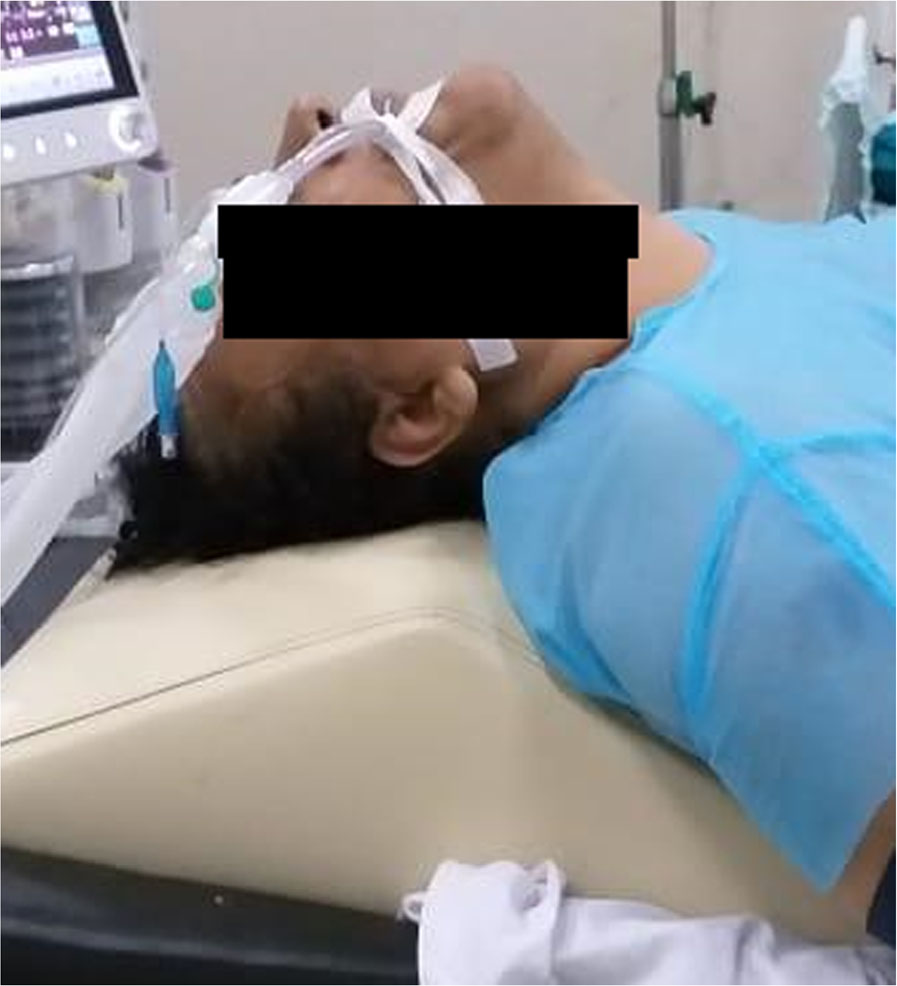
The modified ramped position

Modified ramped position: This position was achieved using a special pillow (Hasanin Pillow). The pillow’s height and length were 15 cm and 60 cm (Fig. 3). The shoulders were elevated, and the head was extended to the most possible range to bring the breasts away from the laryngoscope.

Anesthesia was induced using propofol (2 mg/kg), atracurium (0.5 mg/kg), and fentanyl (2 mcg/kg). Ventilation was maintained using face mask for 3–4 min, then, the endotracheal tube (size 7–7.5 mm) was inserted by the same anesthesiologist (HT) using Macintosh blade (size 3). The laryngoscopic view was graded according to the Cormack-Lehane scale [10] without cricoid pressure.

If the laryngeal visualization was not sufficient in the modified-ramped position group, the head was manually elevated to achieve the ramped position. The position of endotracheal tube was confirmed using the capnography. The special pillow was removed after confirming successful intubation.

### Primary outcome

Incidence of difficult laryngoscopy defined as “failure to insert the laryngoscope in the oral cavity due to large breasts with the need to reposition the patient to insert the laryngoscope”. The term “reposition” means: the need to make further elevation of the patient shoulders by the assistant in order to extend the patient neck and to move the breasts away from the handle of the laryngoscope.

### Secondary outcomes

Time till complete visualization of the vocal cords: defined as the time from starting to handle the laryngoscope till visualization of the vocal cords.

Time of endotracheal intubation: time from starting to handle the laryngoscope till confirmation of the endotracheal tube position by capnography.

Cormack-Lehane [10] grade of vocal cord view (with and without cricoid pressure) as follows:

- Grade I: full visualization of the vocal cords.

- Grade II (a): partial visualization of the vocal cords.

- Grade II (b): only the posterior part of the vocal cords or the arytenoid cartilages are visualized.

- Grade III: only epiglottis is visualized.

- Grade IV: epiglottis is not visualized.

Incidence of relatively difficult mask ventilation: defined as the need of high force and/or oral airway insertion for maintenance of adequate mask ventilation.

Number of trials for endotracheal tube insertion.

Incidence of hypoxemia (defined as oxygen saturation less than 90%) during the period starting from induction of anesthesia till insertion of the endotracheal tube.

Oxygen saturation every 30 s starting from induction of anesthesia till confirmation of the position of endotracheal tube.

End-tidal CO_2_ reading just after insertion of the endotracheal tube.

Incidence of airway trauma (teeth, lips, and tongue trauma).

### Statistical analysis

Our primary outcome was the incidence of difficult laryngoscopy. According to a pilot study, we found that the incidence of difficult laryngoscopy in obese females is 80%. We used G power software (3.1.9.2) to calculate a sample size that detects an absolute risk reduction of 40% in the incidence of difficult laryngoscopy. A total number of 54 patients was calculated to have a study power of 80% and alpha error of 0.05. the number was increased to 60 patients to compensate for dropouts.

Statistical package for social science (SPSS) software, version 21 for Microsoft Windows (SPSS inc., Chicago, iL, USA) was used for data analysis. Categorical data were presented as frequencies (%) and analyzed using chi-squared test. Continuous data were checked for normality using the Shapiro-Wilk test and were presented as means (standard deviations) or medians (quartiles) as appropriate. Continuous data were analyzed using the unpaired t-test or the Mann Whitney test as appropriate. Repeated measures were analyzed using the two-way analysis of variance (ANOVA) for repeated measures with post-hoc pairwise comparisons using the Bonferroni test. A *P* value less than 0.05 was considered statistically significant.

## Results

**Fig. 4:**
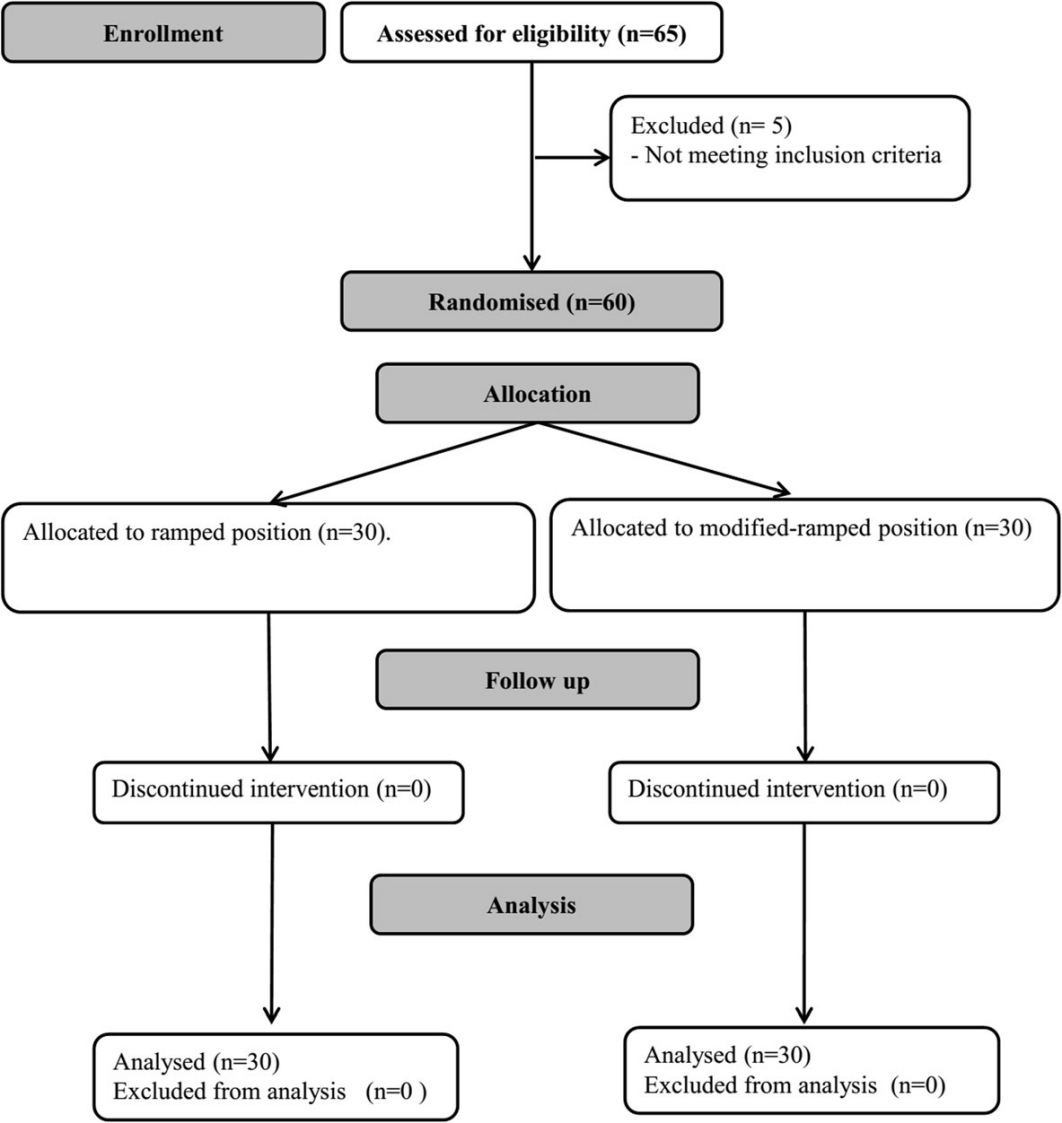
Patient enrollment

Sixty-five patients were screened for eligibility. Five patients were excluded for not meeting our inclusion criteria. Sixty patients were randomized in the study; all of them completed the intervention and were available for final analysis (Fig. 4). Demographic data and baseline characteristics were comparable between both groups (Table 1). The modified-ramped group showed lower incidence of difficult laryngoscopy (3% versus 47%, *p* < 0001), lower incidence of difficult mask ventilation (20% versus 83%, *p* < 0.001), shorter time for glottic visualization (13 ± 3 s versus 17 ± 2 s, *p <* 0.001), and shorter time for endotracheal tube insertion compared to the ramped position (Table 2). The Cormack-Lehane grade of laryngeal view was better in the modified-ramped position (Table 2); however, with cricoid pressure, most of the patients had adequate laryngeal visualization (Cormack-Lehane grade < III) (Table 2). None of the patients in the modified-ramped position needed head elevation to improve the laryngeal view. None of our patients had significant hypoxemia nor airway trauma.

**Table 1:** Baseline characteristics. Data are presented as mean (standard deviation), and frequency (%)

	Ramped group (*n* = 30)	Modified ramped group (*n* = 30)	*P* value
Age (years)	42 (13)	39 (9)	0.26
Body mass index (Kg/m^2^)	41 (6)	43 (6)	0.4
Diabetes	4 (13%)	5 (17%)	1
Hypertension	6 (20%)	9 (30%)	0.55
Snoring	11 (37%)	9 (30%)	0.78
Mallampati score			
Grade I	3 (10%)	8 (27%)	0.35
Grade II	15 (50%)	10 (33%)	
Grade III	10 (33%)	10 (33%)	
Grade IV	2 (7%)	2 (7%)

**Table 2:** Outcomes. Data are presented as mean (standard deviation), median (quartiles), and frequency (%)

	Ramped group (*n* = 30)	Modified ramped group (*n* = 30)	*P* value
Relatively difficult mask ventilation	25 (83%)*	6 (20%)	< 0.001
Difficult laryngoscopy	14 (47%)*	1 (3%)	< 0.001
Time till vocal cord visualization (seconds)	17 (2)*	12 (3)	< 0.001
Time till endotracheal tube insertion (seconds)	42 (3)*	33 (2)	< 0.001
Cormak-Lehane view without cricoid pressure	IIb (IIa-IIb) *	IIa (I-IIb)	0.01
Cormack-Lehane view with cricoid pressure	I (I-IIa) *	I (I-I)	0.03
Cormack-Lehane view without cricoid pressure			
I	5 (17%)	14 (47%)	0.04
II(a)	7 (24%)	8 (27%)	
II(b)	12 (40%)	5 (17%)	
III	6 (20%)	3 (10%)	
Cormack-Lehane view with cricoid pressure			
I	16 (53%)	24 (80%)	0.09
II(a)	11 (37%)	5 (17%)	
II(b)	3 (10%)	1 (3%)	
III			
Number of intubation trials	1 (1,1)	1 (1,1)	1
First end-tidal CO_2_ reading (mmHg)	35.1 (4.4)	34.9 (3.9)	0.88

*denotes statistical significance (*P* < 0.05)

## Discussion

We report that our modification of the ramped position improved the intubation conditions of obese females. This was demonstrated by the better laryngeal visualization, the less need of repositioning, and the shorter intubation time in the modified-ramped position compared to the ramped position.

The original ramped position, which is achieved by elevation of the patient’s head whilst keeping the face in horizontal position, had been described to facilitate airway management of obese patients. In obese patients, there is increased fat deposition in the chest wall, especially in the back, which consequently increases the antero-posterior chest diameter. Therefore, application of the ordinary sniffing position, the recommended position of laryngoscopy, is usually difficult in obese patients. This high chest/head ratio in obese individuals would result in a lower head position when the patient lies flat; thus, the ramped position was proposed to overcome this problem. Collins et al. were the first authors who reported that the ramped position is superior to the sniffing position in morbidly obese patients in terms of laryngeal view; however, they did not report a major difference in the difficulty of intubation [5]. Since then, the studies which compared the ramped position and the sniffing position showed relatively conflicting results. The ramped position was proved superior to the sniffing position in both obese, non-obese populations [11]; and in patients with expected difficult intubation [12]. Semler et al. had, surprisingly, reported different results which favored the sniffing position over the ramped position in 260 critically ill patients [6]. Therefore, further research had been suggested to reach the proper intubating position [7, 8].

In our patients, we introduced a novel modification on the ramped position by the aid of a special pillow. Our modification achieved more neck and head extension than that of the ramped position. This position was hypothesized to 1- Facilitate the insertion of laryngoscope into the oral cavity 2- Improve the mask ventilation. 3- Improve the grade of laryngeal view.

Insertion of the laryngoscope in the mouth cavity is usually difficult in obese females [9]. We reported that patients in modified-ramped position showed easier laryngoscopy and less need for patient reposition. Performing neck extension in the modified-ramped position gave more space for the handle of the laryngoscope away from the sternum and the breast of the patient.

We reported that mask ventilation was easier in the modified-ramped position; this is most probably explained by the easier movement of the jaw when the neck is in extension; whilst, the accumulated fat in the neck and the lower face impaired the jaw movement when the head is in the horizontal plane in the ordinary ramped position. Moreover, when the physician pulls the patient jaw upwards with head in the tilted position, this moves the jaw in 2 directions (anterior and caudal); this would provide better airway patency than moving the jaw in 1 direction (anterior) only when the head in horizontal in the ramped position.

The impact of the patient position on the grade of laryngeal view is a principal factor during comparison of different positions. We had no data about the Cormack-Lehane grade in the modified-ramped position. Therefore, we suggested that manual mobilization of the head would be performed as a rescue maneuver in case of difficult visualization of the glottis; however, we found that, the laryngeal view was better in modified-ramped position and the planned rescue maneuver was not needed in any patient. Proper visualization of the laryngeal view is based in alignment of oral, pharyngeal, and laryngeal axes; this is classically achieved in the sniffing position. The use of the ramped position for improving the laryngeal visualization, although widely applied, is still controversial. The proper alignment of three airway axes was confirmed in the sniffing position using magnetic resonance imaging [4]; however, in the ramped position, the alignment of the three axes is only a theoretical assumption [5] without similar magnetic resonance imaging confirmation. Semler et al. had demonstrated that the ramped position might worsen the laryngoscopic view and increase the number of intubation attempts compared to the sniffing position [6].

Proper position of the head and the neck is an important step for successful laryngoscopy and endotracheal intubation. Airway management in obese patients is relatively challenging due to accumulated fat deposition in the airway that might impair adequate ventilation and visualization of the larynx; furthermore, insertion of the laryngoscope in the oral cavity might also be difficult due to accumulated fat in the anterior chest wall and breasts. Obese patients commonly have restrictive lung disorders which impair their tolerance to any delay in endotracheal intubation. We provided a novel modification for the ramped position which is easily achieved using a simple pillow which provided good space for the handle of the laryngoscope without impairment of the laryngeal visualization. The modified-ramped position would help to avoid the hazards of re-positioning of the patient which is common in obese females; and would consequently avoid unwarranted delay in the endotracheal intubation process.

Our study had some limitations: 1- It is a single center study. 2- Our methodology did not enable blinding of the physician. 3- We investigated our approach in elective, stable patients. We need to confirm its benefits in emergency endotracheal intubation.

## Conclusion

In conclusion, the modified-ramped position provided better intubating conditions, improved the laryngeal view and eliminated the need for repositioning of obese female patients during insertion of the laryngoscope.

## Data Availability

The datasets used and/or analyzed during the current study are available from the corresponding author on reasonable request.

## Abbreviations

ANOVA: Analysis of variance
SPSS: Statistical package for social science
